# Potential Natural Products for Alzheimer’s Disease: Targeted Search Using the Internal Ribosome Entry Site of Tau and Amyloid-β Precursor Protein

**DOI:** 10.3390/ijms16048789

**Published:** 2015-04-20

**Authors:** Yun-Chieh Tasi, Ting-Yu Chin, Ying-Ju Chen, Chun-Chih Huang, Shou-Lun Lee, Tzong-Yuan Wu

**Affiliations:** 1 Graduate Institute of Life Sciences, National Defense Medical Center, Taipei 11490, Taiwan; E-Mail: yun9350751@hotmail.com; 2 Department of Bioscience Technology, Chung Yuan Christian University, Taoyuan 32032, Taiwan; E-Mails: tychin@cycu.edu.tw (T.-Y.C.); assam217@yahoo.com.tw (Y.-J.C.); 3 New Bellus Enterprise Co., Ltd., Tainan 72042, Taiwan; E-Mail: john@newbellus.com.tw; 4 Department of Biological Science and Technology, China Medical University, Taichung 40402, Taiwan; 5 Program and Center of Nanoscience Technology, Chung Yuan Christian University, Taoyuan 32032, Taiwan

**Keywords:** memantine, amyloid precursor protein, tau, Alzheimer’s disease, internal ribosome entry sites, bi-cistronic

## Abstract

Overexpression of the amyloid precursor protein (APP) and the hyperphosphorylation of the tau protein are vital in the understanding of the cause of Alzheimer’s disease (AD). As a consequence, regulation of the expression of both APP and tau proteins is one important approach in combating AD. The APP and tau proteins can be targeted at the levels of transcription, translation and protein structural integrity. This paper reports the utilization of a bi-cistronic vector containing either APP or tau internal ribosome entry site (IRES) elements flanked by β-galactosidase gene (cap-dependent) and secreted alkaline phosphatase (SEAP) (cap-independent) to discern the mechanism of action of memantine, an *N*-methyl-d-aspartate (NMDA) receptor antagonist. Results indicate that memantine could reduce the activity of both the APP and tau IRES at a concentration of ~10 μM (monitored by SEAP activity) without interfering with the cap-dependent translation as monitored by the β-galactosidase assay. Western blot analysis of the tau protein in neuroblastoma (N2A) and rat hippocampal cells confirmed the halting of the expression of the tau proteins. We also employed this approach to identify a preparation named *NB34*, extracts of *Boussingaultia baselloides* (madeira-vine) fermented with *Lactobacillus* spp., which can function similarly to memantine in both IRES of APP and Tau. The water maze test demonstrated that *NB34* could improve the spatial memory of a high fat diet induced neurodegeneration in apolipoprotein E-knockout (ApoE^−/−^) mice. These results revealed that the bi-cistronic vector provided a simple, and effective platform in screening and establishing the mechanistic action of potential compounds for the treatment and management of AD.

## 1. Introduction

Alzheimer’s disease (AD) is considered as the most common neurodegenerative malady in the modern but senile society [1]. This condition is characterized primarily by dementia which afflicts an estimated 35.6 million people worldwide and the numbers are estimated to be doubling every 20 years [2,3]. Anatomical dissections and analyses of the brains of AD patients have led to the identification of two hallmarks defining the neuropathological characteristics of this disease: neuritic plaques and neurofibrillary tangles (NFTs). Under the electron microscope, abnormal amyloid-like filaments were found in the plaques and neurofibrillary tangles [4,5]. The specific sites of plaques and tangles are different: plaque filaments are extracellular but most of the tangled filaments are present intracellularly; deposited in nerve cell bodies, as well as in neurites of neuron. The major molecular compositions of the plaques and tangles are also different: amyloid-β (Aβ) peptide [6] is the major plaque component while the tau protein [7] is the major tangle component. The 40–42 amino acid Aβ peptide is derived from the sequential cleavage of amyloid precursor protein (APP), a type 1 transmembrane protein, by two proteases, β- and γ-secretase [1,2]. Tau protein, on the other hand, is one of the main neuronal microtubule-associated protein and functions importantly in the modulation of microtubule organization during morphogenesis and process outgrowth in neurons [8]. Transgenic mouse models of AD that target the APP and tau genes also confirmed the pathogenetic factors [9,10,11,12]. All these studies indicate that the control of the expression of APP and tau in the brain may be a good target for drugs that can potentially be used in the treatment of AD.

Aside from the canonical cap-dependent model of recognition and ribosomal scanning, there is an alternative method of translation initiation, named cap-independent translation, as first described in members of the family *Picornaviridae* [13,14] and subsequently for a growing subset of cellular mRNAs [15,16]. In the cap-independent mechanism of translation initiation, ribosomes are recruited to the mRNA by RNA structural elements called internal ribosome entry sites (IRESes) [17]. Since it has been shown that many cellular mRNAs contain IRESes, it is likely that up to 10% of all mRNAs have the capability to initiate translation by the cap-independent mechanism [18,19]. It is also apparent that genes involved in a diverse range of cellular activities, including proliferation, growth and apoptosis employ this alternative mechanism resulting in the consideration of internal initiation through IRES as an important cellular mechanism and not just a specialized viral strategy [15]. Recent studies demonstrated that the APP mRNA may be translated through an IRES wherein APP mRNA was found to be one of the several mRNAs which may remain associated with polyribosomes during mitosis, when cap-dependent translation initiation is greatly diminished [20]. Interestingly, it has also been reported that the 5' leader in the human tau mRNA contains an IRES and that IRES-dependent translation plays a significant role in the generation of the tau protein [21].

Memantine is a US Food and Drug Administration-approved, uncompetitive *N*-methyl-d-aspartate (NMDA) receptor antagonist and reduces clinical deterioration in moderate to severe AD. Preclinical evaluations regarding the use of memantine as an NMDA receptor antagonist were reviewed and reported extensively [22,23,24,25,26]. In addition, memantine is also capable of halting and reversing the protein phosphatase (PP)-2A inhibition-induced abnormal hyperphosphorylation of tau/neurofibrillary degeneration [27], and protects the neurons from microglial-inflammatory responses that result to cell death [28]. Recently, our laboratory linked NMDA receptor antagonists, amantadine and memantine [22], with the down-regulation of the IRES of enterovirus 71 and encephalomyocarditis virus [29,30].

In this study, we report that the NMDA receptor antagonist, memantine can suppress the expression of neuronal APP and tau proteins through the novel cap-independent translational initiation mechanism. Based on this observation, we also employed the IRESes of APP and Tau as the potential targets for AD to screen *Lactobacillus* spp. fermented traditional Chinese herbs and identified a preparation that can inhibit the translational activity of the Tau IRES. The water maze test demonstrated that this fermented preparation could improve the spatial memory of high-fat diet (HFD) induced neurodegeneration in ApoE^−/−^ mice.

## 2. Results

### 2.1. The Amyloid Precursor Protein and the Tau IRESes Construct

The expression of APP and tau proteins has been demonstrated in a number of reported studies to be mediated by IRES, an atypical translational initiation mechanism, aside from the conventional cap-dependent translation initiation [20,21].

**Figure 1:**
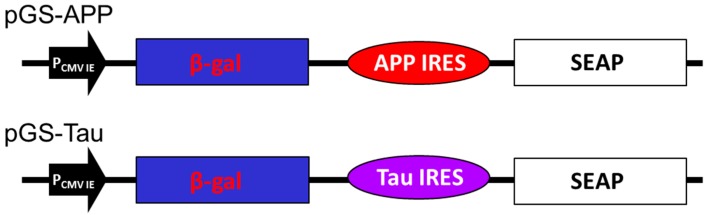
Construct of pGS-APP and pGS-Tau. The bi-cistronic vectors contain the genes for beta-galactosidase (β-Gal) and secretory alkaline phosphatase (SEAP), IRES element from the APP or the Tau genes, APP IRES and Tau IRES, respectively. P_CMV IE_ is the promoter of human cytomegavirus immediately early promoter.

A previous study had shown that amantadine can inhibit the translation activity of IRES derived from HAV, enterovirus 71 or encephalomyocarditis virus [30]. It is interesting to note that the chemical structure of amantadine is similar to memantine, a therapeutic drug for moderate to severe AD and both are tricyclic symmetric amines. The plasmid pTriEx4, containing either the genes for the β-galactosidase or the secreted alkaline phosphatase, were used for the construction of the two bi-cistronic vectors as shown in Figure 1 (as described in the Experimental section). The bi-cistronic vectors were generated by inserting either the APP (pGS-APP) or Tau (pGS-Tau) IRES of DNA fragments in between the β-galactosidase (β-Gal) and secreted alkaline phosphatase (SEAP) reporter genes (Figure 1).

### 2.2. The Tissue Tropism of APP and Tau IRESes and the Effect of Memantine on APP and Tau IRESes

The ability of IRESes to initiate translation varies greatly in cells of different origin. Therefore, we presumed that both APP and tau IRESes could drive more efficient cap-independent translation in neuron-like cells, *i.e.*, N2A, than in non-neuronal cells such as COS-1 and CHO cells. To test this presumption, we performed transient transfection assays on different cell lines using the plasmids listed in Figure 1.

**Figure 2:**
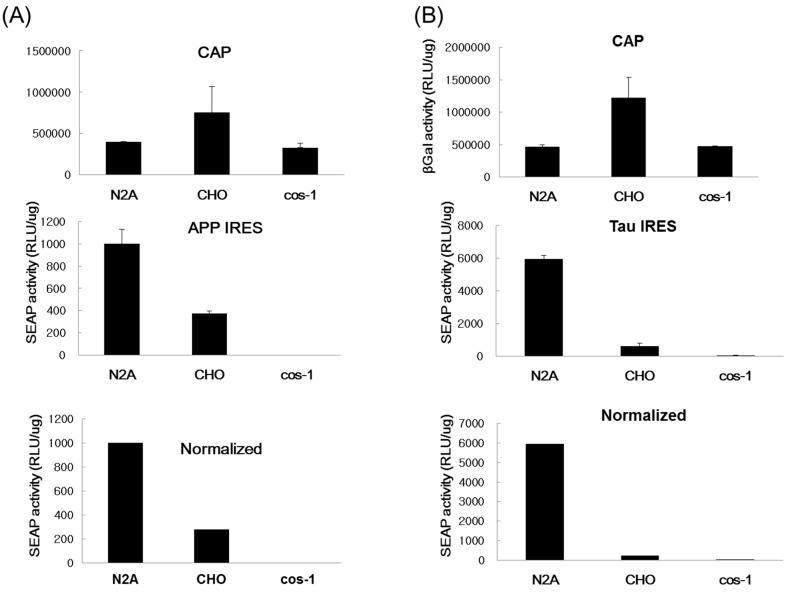
Determination of cell tropism of the APP and tau IRES. Transient transfection with the corresponding plasmid was performed on mammalian cells (~9 × 10^4^ cells/well) of different types using Lipofectamine 2000. Cap-dependent mechanism of translation was assessed by β-galactosidase activity in the cell lysate while the IRES-dependent mechanism of translation was ascertained through the SEAP activity in the cell medium. Normalization was carried out by β-galactosidase assay. (**A**) Comparison of the APP IRES and (**B**) Tau IRES activity in mouse N2A, CHO and COS-1 cells.

It was observed that the CHO cells gave a two-fold β-Gal activity upon transfection with the pGS-APP plasmid, signifying an efficient transfection of the plasmid DNA as compared to the N2A and the COS-1 cells (Figure 2A). However, considering the activity of the reporter protein SEAP, it is apparent that the N2A cells showed a significantly increased secreted alkaline phosphatase activity that is three times the measured activity in CHO cells, whereas the SEAP activity was not observed in COS-1 cells. After normalizing the SEAP activity using the β-Gal assay, results clearly indicated that APP IRES favored the cap-independent translation specifically to the N2A neuron-like cells. Similar data were obtained after cell lines were transfected with the plasmid containing the tau IRES (Figure 2B).

**Figure 3:**
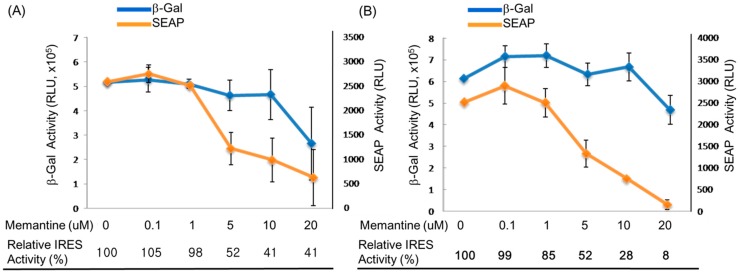
Effect of various concentrations of memantine on mouse N2A neuroblastoma cells. The N2A cells (~9 × 10^4^ cells/well) were seeded onto a 24-well plate prior to transfection. Lipofectamine (2 μL) was used to transfect the plasmid DNA (1 μg) into the neuroblastoma cells. At 24 h post treatment with memantine, the culture medium from each well was harvested, the cells were lysed, and subsequent SEAP and β-galactosidase assay were done, respectively. The effect of memantine on (**A**) AβPP IRES and (**B**) tau IRES, as evaluated by beta-galactosidase (cap-dependent) and SEAP (IRES-dependent) activities in murine neuroblastoma (N2A) cells.

It is conspicuously seen in this result that the tau IRES is significantly more functional in the N2A cells since the other cell lines tested gave a very little or no SEAP activity at all. In addition, the tau IRES was also found to be more functional than the APP IRES in N2A cells considering the six-fold increase of the SEAP activity in the cell medium of the N2A cells transfected with the plasmid containing the tau IRES. The results presented herein were consistent with the tissue tropism [31] of either APP or Tau IRESes [32]. More interestingly, data revealed that memantine was able to inhibit the IRES activity of APP or tau without interfering with the cap-dependent translation. At a memantine concentration of 5 μM, a significant decrease equivalent to 52% relative APP (Figure 3A) and tau (Figure 3B) IRES activity was observed after 24 h treatment. This result implied that memantine, comparable to amantadine [30] can also act as an inhibitor of IRESes and on this note, a regulator of the translation of the APP and tau proteins. This in turn further supports the previously reported hypothesis [29] that memantine has a dual action in the management and treatment of Alzheimer’s disease. Memantine does not only obstruct the excitotoxicity of NMDA receptors [33,34,35] but also halts the expression of APP and tau proteins through IRES. The inhibition of the APP IRES and tau IRES by memantine might indicate the diminished Aβ production and tauopathies as well as an antagonist of NMDA receptors in AD.

### 2.3. Effect of Memantine on Expression of the Amyloid Precursor Protein and Tau in Neuronal Cells

Aggregated Aβ affects neurons and induces NFT formation and neuronal loss, eventually leading to dementia [36]. In a number of cell culture based studies on the assessment of the pharmacological effects of memantine, it was revealed that memantine exerted significant activity at a concentration range between 1 and 20 μM [35,37,38].

**Figure 4:**
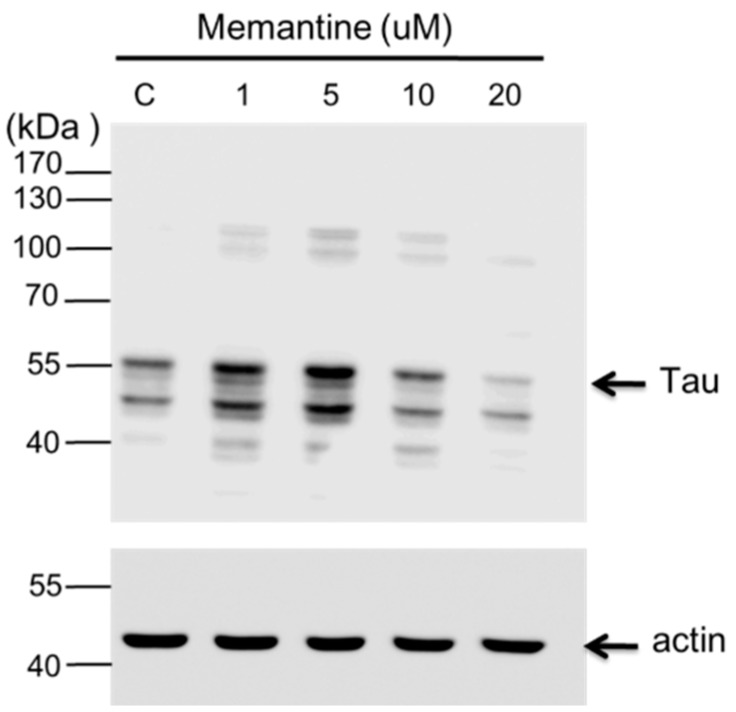
Western blot analysis showing the effect of memantine on tau protein expression in the N2A cell lysate. The neuroblastoma cells (~9 × 10^4^) were treated with different concentrations of memantine (1, 5, 10 and 20 μM). The PVDF membrane was exposed to anti-tau-1 (clone PC1C6) antibody to detect the endogenous tau protein from N2A cells. Each lane corresponds to the concentration (1–20 μM) of memantine treatment; Lane C is for the untreated cell lysate. Tau protein is visible as bands between the 40 and 55 kDa regions. Molecular weight marker is shown left most. β-Actin was used as a loading control.

**Figure 5:**
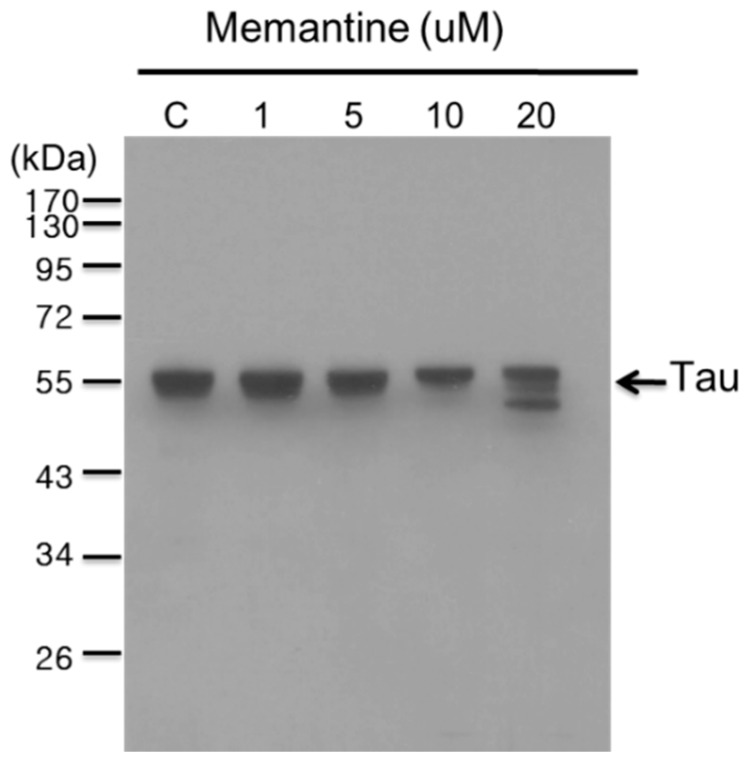
Western blot analysis showing the effect of memantine on tau protein expression in the rat hippocampal cell lysate. The hippocampal cells (5 × 10^4^/cm^2^) were treated with different concentrations of memantine (1, 5, 10 and 20 μM). The PVDF membrane was incubated with anti-tau-1 (clone PC1C6) antibody to identify the endogenous tau protein from N2A cells. Each lane corresponds to the concentration (1–20 μM) of memantine treatment; Lane C is for the untreated cell lysate. Tau protein is visible as bands in between the 43 and 55 kDa regions. Molecular weight marker is shown left most.

In this study, concentrations: 1, 5, 10 and 20 μM of memantine were used to treat mouse N2A (Figure 4) and rat hippocampal (Figure 5) neuronal cells and to monitor the expression of tau and APP proteins. Tau is a single copy gene in both humans and rats that goes through alternative message splicing resulting in multiple isoforms in adults [39]. These isoforms are post-translationally modified via phosphorylation [40]. The monoclonal antibody for Tau-1 used in this study identifies an amino acid sequence from 192 to 204 in humans and 180–198 in rats when all of the four serine residues are unphosphorylated [41]. Western blot analysis confirmed the presence of tau-1 proteins from both neuronal (mouse N2A and rat hippocampus) cell lysates treated with various concentrations of memantine (1, 5, 10 and 20 μM) as revealed by bands at around 52–68 kDa. It is clearly depicted in Figure 4 that memantine was able to halt the expression of the tau-1 protein in the murine model as seen through the observed faint bands at approximately 52–55 kDa corresponding to different isoforms of mouse tau protein [42] after treatment with 10 μM memantine. This is in agreement with the result obtained upon treatment of the rat hippocampal cells with memantine wherein the appearance of a diminished bandwidth along ~55 kDa at a concentration of 10 μM signifies the stalling of the tau protein expression (Figure 5). Furthermore, densitometer scan of gel bands in Figure 5 indicated that expression levels of the tau protein were inhibited about 25% with 10 or 20 µM of memantine added in the culture medium of hippocampal cells. It is also evident in Figure 5 that dephosphorylation of the tau-protein may have occurred after it was treated with 20 μM memantine as shown by the two visible bands at lower molecular weight regions. The experimental results therefore provide initial evidence for the potential use of the IRES of the tau protein as a novel target for the screening of compounds for the treatment of AD and other tauopathies.

We also evaluated the effect of memantine on the expression of APP by Western analysis and revealed a minimal dose-dependent increase in the intracellular APP as the concentration approaches 10 μM. It is noteworthy to mention that at a concentration of 20 μM, the intracellular APP was increased markedly (data not shown). This result is consistent with the study by Ray *et al*. [43], where a significant increase in intracellular APP levels with a 20 μM dose of memantine in human neuroblastoma cells was reported. However, they also show that memantine treatment decreases levels of secreted APP and Aβ peptide in their report [43]. These results may imply that memantine can enhance APP protein expression levels through an uncharacterized mechanism, although memantine can inhibit APP IRES activity. Such a novel property of memantine demands further study to clarify conflicting results and define its beneficial effect on AD.

### 2.4. Identification of NB34 as a Potent Inhibitor of Tau IRES

**Figure 6:**
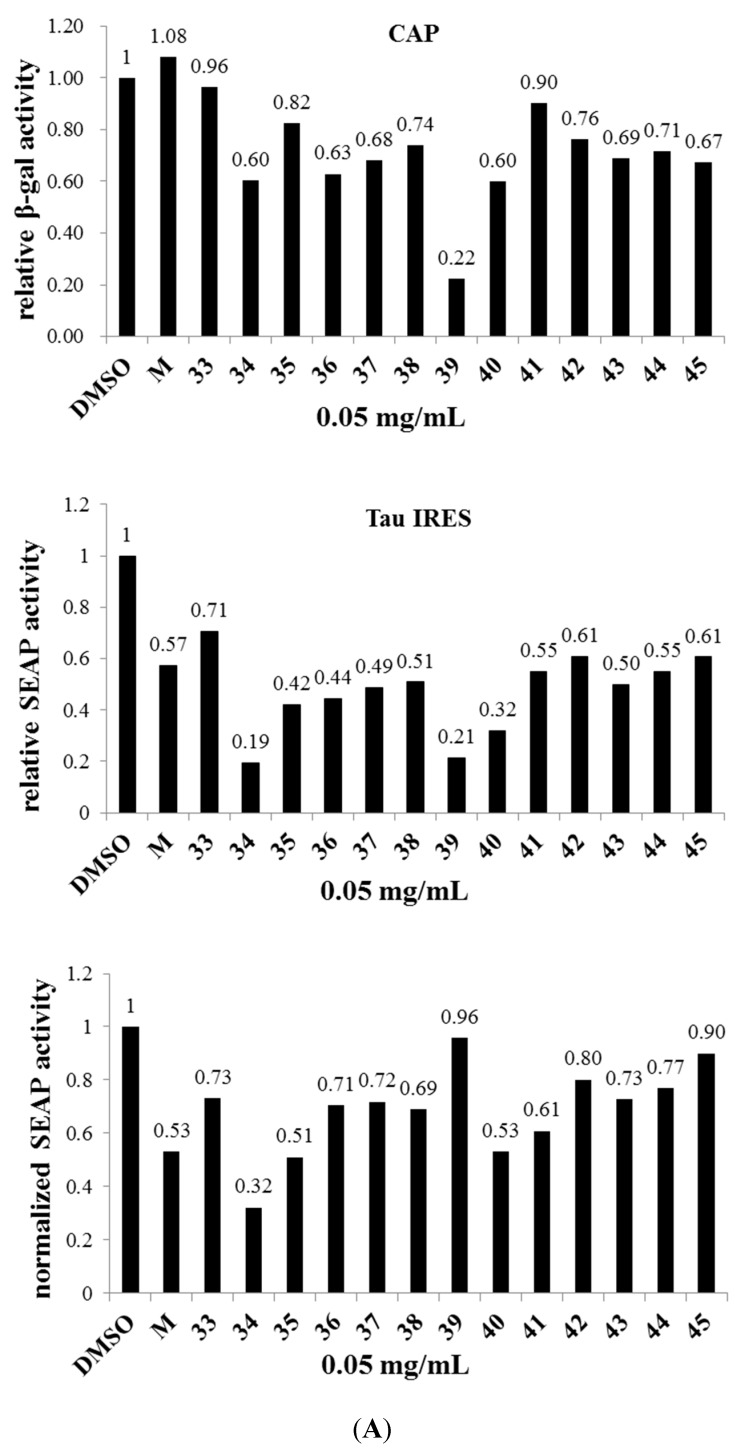

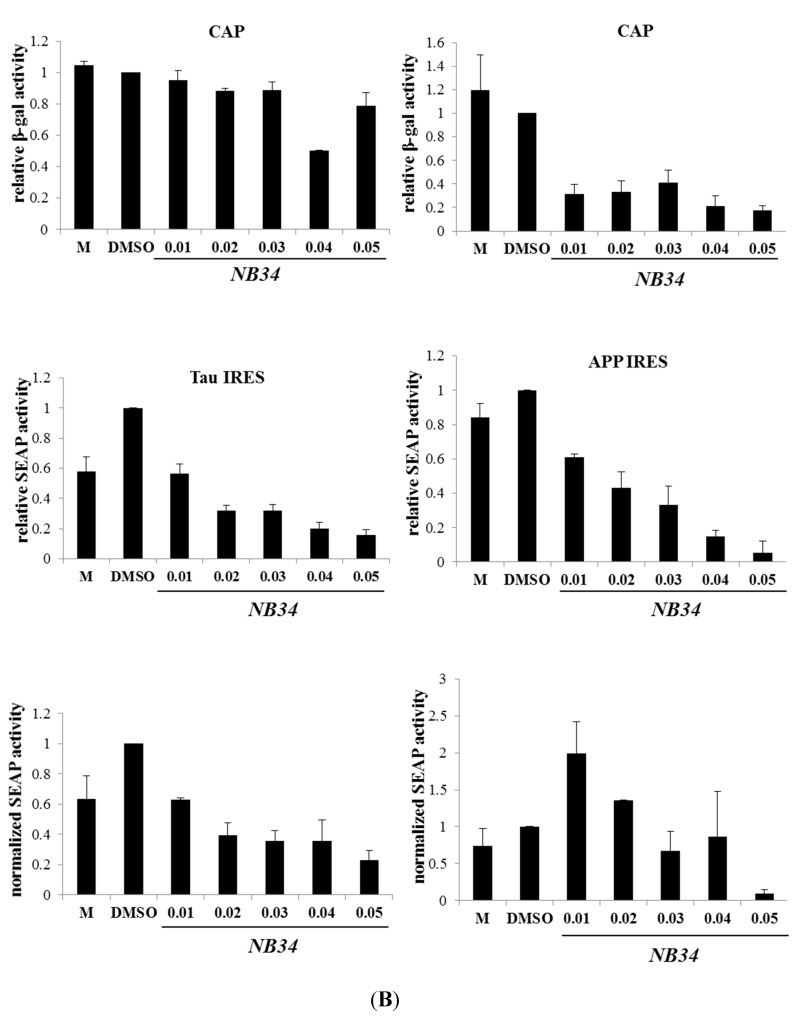
Identification of *NB34* as an inhibitor for Tau IRES in N2A cells. (**A**) The N2A cells (~9 × 10^4^ cells/well) were seeded onto a 24-well plate prior to transfection. Lipofectamine (1 μL) was used to transfect the pGS-Tau plasmid DNA (1 μg) into the neuroblastoma cells. At 24 h post treatment with various preparation (0.05 mg/mL) of Chinese herbs fermented by *Lactobacillus* spp., (*NB1-92*, and only the *NB33-45* are shown) the culture medium from each well was harvested, cells were lysed and subsequent SEAP and β-galactosidase assays were made, respectively; (**B**) The dose dependence of *NB34* on translation activity of APP IRES and Tau IRES in N2A cells. M, 0.25 μM memantine.

Mementine can inhibit both APP and Tau IRES, indicating that both the IRES could be potential targets for compound screening for AD. Traditional Chinese medicines (TCM) have been widely investigated for the treatment of Alzheimer’s disease [44], and reports indicated that fermentation of these Chinese herbs by microbes, such as *Lactobacillus* spp., could dramatically enhance the concentration of active compounds. Thus, we tried to prepare 92 different preparations that were derived from *Eleutherococcus senticosus*, *Lycium chinense* Miller, *Panax ginseg*, *Curcuma longa*, *Radix notoginseng* and *Gastrodia elata* after fermentation by *Lactobacillus* spp. All the preparations were named NB1 to NB92. Figure 6A shows that the preparation, named *NB34*, could inhibit the Tau IRES mediated translation activity in N2A cells. We further studied the dose response of *NB34* on the Tau IRES and APP IRES activity. Figure 6B shows that the *NB34* could inhibit the translational activity of Tau IRES as low as 0.02 mg/mL, although its effect on translational activity of APP IRES was only obvious at 0.05 mg/mL. Thus, we were interested to investigate whether *NB34* could work as mementine to benefit AD. *NB34* is the product of *Radix notoginseng* fermented by *Lactobacillus* spp., and interestingly, a previous study demonstrated the anti-aging effect of *Radix notoginseng* on cultured neurons of rats with AD [45]. Thus, we tried to evaluate whether this novel *NB34* preparation could be beneficial for memory in mice using the Morris water maze task.

### 2.5. NB34 Inhibits Impairment of Spatial Learning Induced by High Fat Diets during Memory Acquisition in ApoE^−/−^ Mice

ApoE4 allele is recognized as a prominent risk factor for the development of AD in human [46,47]. In mice, ApoE protects against neuropathology induced by HFD and mice deficient in ApoE display disturbances in learning and memory function such as long-term potentiation [43], loss of synapses with age, or cytoskeletal alterations [48,49,50]. Thus, we employed the HFD feeding of ApoE^−/−^ mice to address whether *NB34* could improve the spatial memory of HFD-fed ApoE^−/−^ mice.

**Figure 7:**
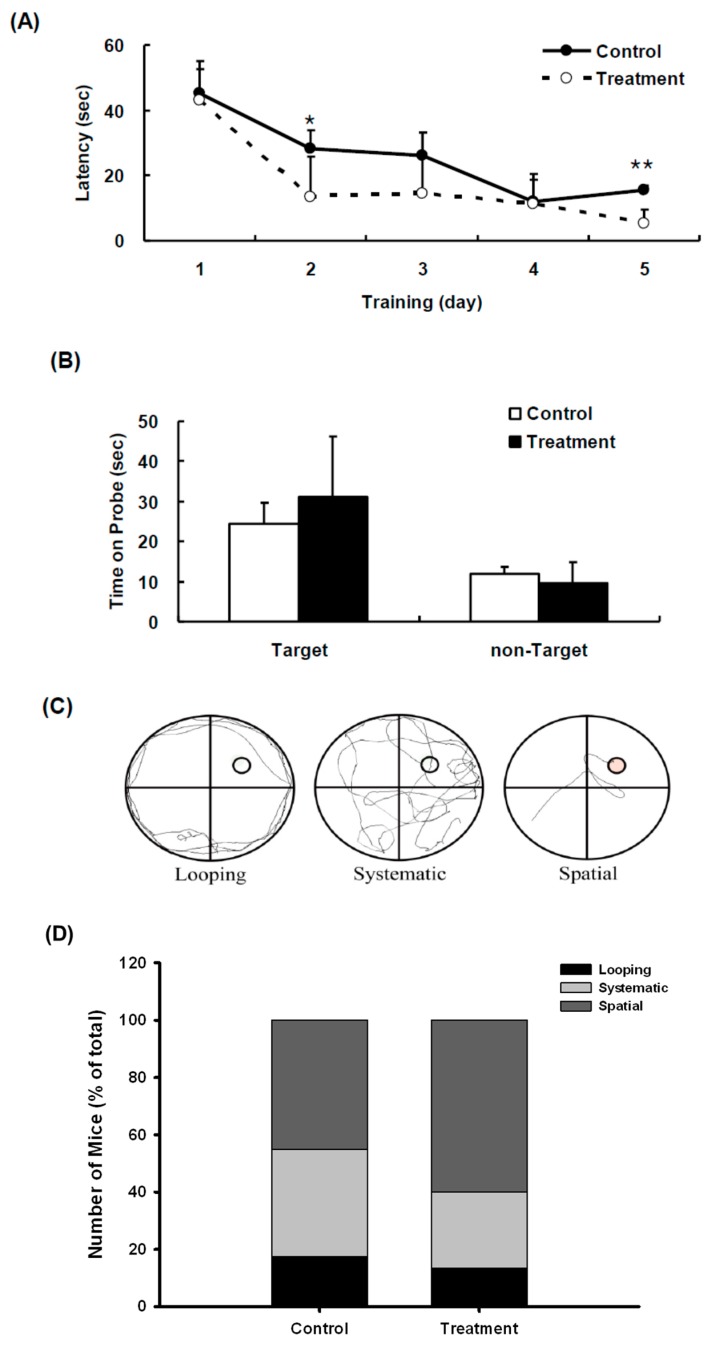
Effects of *NB34* on Spatial memory performance in the Morris water maze (MWM). (**A**) Acquisition trials. The time spent to reach the platform (escape latency). * *p* < 0.05 when compared with the corresponding control groups; ** *p* < 0.005 when compared with the corresponding control groups; (**B**) Probe trial. Probe test performed 24 h after the hidden platform acquisition period. Time spent in the target quadrant was similar in two groups; (**C**) Examples of search strategies; (**D**) Comparison of swimming strategies during acquisition phase of MWM between control and *NB34* treated ApoE^−/−^ mice combine with HFD. Search strategy was examined for first of the 4 trials on different acquisition days.

To assess whether simultaneous intake of *NB34* could reverse HFD-induced learning and memory impairments in ApoE^−/−^ mice, a battery of behavioral tests was conducted. Both groups learned to find the hidden platform during the acquisition phase of training (days 1–5), which was shown by a progressive decrease in latency to reach the platform. *NB34*-treated mice appeared to reach the platform faster than control mice at day 2 (*p* < 0.05; Figure 7A) and day 5 (*p* < 0.005; Figure 7A). However, in the probe trial, there was no significant difference in the time spent in the target quadrant between the two groups (Figure 7B).

### 2.6. NB34 Administration Results in Increased Use of Spatial Search Strategies

To assess the effect of diet on spatial learning, mice were fed diets containing NB34, we also analyzed the respective search patterns shown by the mice to locate the hidden platform on each day of the first acquisition period in the Morris water maze test. We categorized the behavior of individual mice according to the incidence of distinct search strategies, an outcome that is less influenced by locomotion deficits. Figure 7C indicated the individual mouse strategies reveal three learning phases, e.g., spatial strategy, systemic strategy, and looping strategy, following previously published criteria [51,52,53]. Three different search strategies were defined as follows: swimming directly to the correct target quadrant and searching was called the spatial strategy; systematic strategy was defined as searching interior portion of or entire tank; and if more than 70% of the swim trace was outside the circle and swimming around the wall of tank, it was defined as the looping strategy. The search strategies were examined for the first of the four trials on different training days. ApoE^−/−^ mice with NB34 treatment displayed increase spatial type (60% *vs.* 45% from control mice) and reduced looping type (13.3% *vs.* 17.5% from control mice). The mice with spatial strategy for platform indicate better learning behavior than those with looping strategy. Comparison of swimming strategies indicated that *NB34* treated mice had increased spatial strategies for platform, indicating better learning, while control mice, despite having increased systemic approaches to platform, also had increased looping approaches indicating poor learning (Figure 7D).

## 3. Discussion

The bi-cistronic assay is considered the gold standard to define internal initiation of translation and is one of the most widely used methods for testing supposed IRES sequences [31,54,55]. Thus, we constructed bi-cistronic plasmids to monitor the activity of the 5'-UTR of both the APP and tau as well as to verify the neurotropism of these IRESes. Notably, the ribosomes and other components of the translation machinery were also found in the dendritic processes of neurons, although at minimal amounts compared to those found in the cell body [56,57,58]. Previous reports indicated that APP [20,59] and Tau [21] mRNAs can be translated through an IRES. It has been well documented that the APP 5'-leader contains an IRES and showed that IRES-dependent translation is a mechanism by which endogenous APP mRNA is translated [59]. The same is true with the tau protein wherein it exhibits the characteristics of a viral IRES that contains a relatively lengthy 5' leader at 250 bp with a high guanine/cytosine (G/C) content and this IRES in turn functions in the regulation of the synthesis of the tau protein [21]. Therefore, these reports indicate that IRES are good targets for AD treatment due to the reason that the internal initiation of translation of the APP and tau mRNAs is an important mode for the synthesis of both APP and tau, a mechanism which is controlled by conditions that also contribute to AD pathology [59,60]. Based on these observations, we also identified NB34, a preparation of *Radix notoginseng* fermented by *Lactobacillus* spp. that like memantine could inhibit the translational activity of Tau IRES. Interestingly, NB34 could rescue HFD-induced learning and memory impairments in ApoE^−/−^ mice (Figure 7). Although the functional components in NB34 that can inhibit the translational activity of Tau IRES is unknown, the preparation of *Radix notoginseng* fermented by *Lactobacillus* spp. might be developed as a potential health food in the future. Thus, identification of the compound(s) that are responsible for NB34 function in learning and memory of ApoE^−/−^ mice would be critical and necessary in the future.

In our study, the NMDA-receptor antagonist- memantine was shown to inhibit both the APP and tau IRES without restraining the cap-dependent translation when the concentration of memantine is below 10 μM as revealed through the monitoring of the activity of beta-galactosidase in N2A cells (Figure 3A,B). The result of our study is in good agreement with the recent study of Ray and co-workers [61] where human neuroblastoma SK-N-SH cells treated with 10 μM memantine decreased the measured levels of secreted total APP (sAβPP), APPα isoform and Aβ_(1–40)_ in a time dependent manner for up to 24 h.

Result of transient transfection studies in different cell lines clearly implied the preferential activity of both APP and tau IRESes on neuronal cells rather than on CHO and COS-1 cells (Figure 2A,B), which could be attributed to the tissue tropism of the APP and tau IRESes. Anti-Aβ (β-amyloid) therapy has been a standard approach toward the development of treatment against AD. The secretion of Aβ in AD leads to the production of highly reactive oxygen species (ROS) and mitochondrial defects [62]. Thereby, the continuous secretion of Aβ together with high levels of oxidative stress leads to a cascade of events resulting in the degeneration of neurons and eventually cell death. Memantine prevented Aβ-induced memory impairment in rats that received bilateral microinjections of aggregated Aβ_1-40_ into the (*Cornu Ammonis*, CA) CA1 and CA3 subfields of the rat hippocampus has been reported. Nakamura and co-workers [63] reported that subcutaneous infusion of memantine at doses of 10 and 20 mg/kg/day for six weeks starting 24 h before aggregated Aβ_1-40_ significantly prevented learning deficits and hippocampal damage in rats. Here, we report the effect of the NMDA-receptor antagonist—memantine, on the expression of both APP and tau proteins in neuronal cells. In the progression of the debilitating Alzheimer’s disease, excitotoxicity is considered as a contributing factor specifically on the induction of neuronal cell death [33]. The over-activation of the NMDA type glutamate receptor increases the ability of the calcium ions (Ca^2+^) to enter the cytosol, thus acting as one of the culprits of cell injury, damage and even cell death (details are discussed in the review of Lipton [64]). Many studies *in vitro* also suggest that glutamate receptors specifically the NMDA receptors contribute to neuronal toxicity produced by the accumulation of the β-amyloid peptide [65,66].

In our present study, memantine is also demonstrated to down regulate the expression of tau, consistent with our previous report [29]. The biological activity of tau is controlled by the degree of its phosphorylation. The abnormally hyperphosphorylated tau sequesters the microtubule associated proteins (MAP-1 and MAP-2) or normal tau, resulting in the breakdown of the microtubule networks, and subsequent neurofibrillary degeneration and other tauopathies may develop [67]. Studies have shown that memantine is capable of modulating the signaling pathways of the protein phosphatase-2A, an enzyme responsible for the phosphorylation of tau [27,68].

Interestingly, no direct evidence indicated the higher activity of APP and Tau IRES result in higher release of Aβ and tau extracellularly and intracellularly, respectively. However, Han *et al.*, analyzed published data on the AD blood transcriptome and revealed that the perturbation of cellular functional units could lead to the upregulation of cellular IRES activity [69]. This *in silico* study indicated that aberrant expression of APP and Tau IRES might increase the release of Aβ and tau extracellularly and intracellularly, respectively. Two unexpected observation are also revealed in Figure 4. First, there were two faint but clear bands around 100KDa upon addition of memantine in the N2A cell lysate. Previous studies demonstrated that tau multimers with apparent molecular weights of ~140 and ~170 kDa are in fact tau dimers of 120 and 130 kDa, based on Bis-Tris or Tris-acetate SDS-PAGE migration [70]. However, the bands around 100 kDa were less than 140 kDa, although the major band that represent the monomeric tau proteins were around ~43 and ~60 kDa, consistent with the alternative splicing forms of tau proteins. Thus, these extra faint bands might not be the dimers of Tau proteins. However, we did not exclude the possibility that the faint bands were dimers of Tau protein at present. Second, densitometry analysis using image J on the gel in Figure 4 revealed that the three gel bands observed between 40 and 55 kDa increased about 65% and 85%, respectively, in the presence of 1 and 5 μM of memantine when compared with the control. The control and 10 μM lanes show no significant difference and there was a 35% decrease between 20 μM and the control. These results might imply that while memantine can inhibit the IRES activity of Tau, it may enhance the expression of Tau proteins within the neuronal cells when the concentration of memantine is lower than 10 μM, although 20 μM of memantine indeed suppressed the expression of Tau protein in N2A cells. Further studies to clarify these unusual results may be beneficial for an understanding of the action mechanisms of mematine on AD.

Based from the results obtained in our study, memantine can be used as a potential IRES-dependent translational inhibitor since it is capable of stalling the activity of tau-IRES, leading to diminished tau protein expression. Thus, the action of memantine on AD may be due to its inhibition of NMDA receptor-induced excitotoxicity as well as through the inhibition of IRES-mediated translation. Our findings also offer a facile method for screening biologically important compounds that may play a significant role in arresting the development of Alzheimer’s disease. However, the precise mechanism on how memantine inhibits the IRES of both tau and APP remains elusive. These IRESes may form specific RNA structures that operate as aptamers or molecular switches in response to the direct binding of various compounds to regulate and control gene translation. Further studies are yet to be conducted on an in-depth understanding of the mechanism of the IRES-mediated translation in the pathogenesis of AD.

## 4. Experimental Section

### 4.1. Culturing of Cells, Plasmids Construction and Transfection Studies on Mammalian Cells

The cell lines used in the experiment were COS-1 (African green monkey kidney fibroblast-like cells; Bioresource Collection and Research Center (BCRC), Taiwan; 60002), CHO (Chinese hamster ovary cells; BCRC, 60006) and N2A (Mouse neuroblastoma; BCRC, 60026). The COS-1 and CHO cells were grown in Dulbecco’s medium (DMEM) (Invitrogen, Carlsbad, CA, USA) while the N2A cells were grown in Minimum Essential Medium (MEM) (Invitrogen) both supplied with 10% fetal bovine serum. The plasmid pUC57 (NCBI No. NM016835) containing the tau IRES DNA fragment was synthesized by PROTECH Technology Company, Taiwan. The 240 bp tau IRES was cut from pUC57 by enzymatic digestion with *Not*1 and cloned into the *Not*1 treated plasmid-pGS-EMCV [69] to replace the EMCV IRES fragment. The AβPP IRES DNA fragment was amplified by PCR using two pairs of primers: (1 Forward: ATTGCGGCCGCAGTTTCCTCGGCAGCGGTAGGCGAGAGCACGCGGAGGAGCGTGCGC; 2 Reverse: TCTGCCCGCGCCGCCACCGCCGCCGTCTCCCGGGGCCCCCGCGCACGCTCCTCCGCGT; 3 Forward: TGGCGGCGCGGGCAGAGCAAGGACGCGGCGGATCCCADTCGCACAGCAGCGCACTC; 4 Reverse: TATGCGGCCGCCGCGACCCTGCGCGGGGCACCGAGTGCGCTGCTGTGCGA). The restriction sites in *Not*1I are underlined. The AβPP IRES containing DNA fragment was digested with *Not*1 and cloned into the *Not*1 treated plasmid-pGS-EMCV [71] to replace the EMCV IRES fragment. The plasmids were generated as pGS-APP and pGS-Tau, respectively (Figure 1). In each plasmid, the IRES element from the genes of either APP or tau was flanked correspondingly by the reporter genes β-galactosidase and secreted human alkaline phosphatase (SEAP). Prior to transfection, cells from the corresponding cell line were seeded onto a 24-well plate at a density ~9.0 × 10^4^ cells/well. The cells were washed repeatedly with serum-free medium to remove all traces of sera. Plasmids were then transfected into the respective cell line using Lipofectamine 2000 reagent (Invitrogen). Briefly, the plasmid DNA (1 μg) was diluted with serum-free either DMEM or MEM (50 μL), then the Lipofectamine 2000 reagent (1 μL) was added and allowed to form the complexation product for 15~20 min, followed by transfection to the respective cells and incubated at 37 °C with 95% air and 5% CO_2_. After five hours, the transfection medium was removed, the adherent cells were washed with PBS and then replaced with a fresh medium (with 10% FBS and antibiotics) and the corresponding memantine dosage.

### 4.2. IRES Reporter Assay

Memantine solutions were prepared at various concentrations (0.1, 1, 5, 10 and 20 μM) by dilution with DMEM or MEM to treat the transfected COS-1, CHO and N2A cells. Twenty-four hours post-addition of memantine, the cell culture medium from each well was pipetted out and was kept frozen at −20 °C until used for SEAP activity measurement using BD Great EscApe SEAP detection kit (Clontech, Palo Alto, CA, USA). Subsequently, remaining cells were lysed for 10 min in 120 μL of lysis buffer (Cytobuster™ Protein Extraction Reagen; Novagen, Madison, WI, USA) and was subjected to centrifugation at 12,800 rpm for 10 min the lysate supernatant was then assayed for β-galactosidase activity using Luminiscent β-Galactosidase Detection Kit II (BD Biosciences, San Jose, CA, USA). The chemiluminescence intensity (relative light units, RLU) and was measured with Mithras LB 940 (Berthold Technologies, Wildbad, Germany) chemical luminescence counter.

### 4.3. Western Blot Analysis of the APP and Tau Proteins in Neuronal Cells

Primary hippocampal neurons were dissociated from the rat fetuses at the 18th embryonic day according to the procedures previously described [72,73]. Inhibition of the expression of the tau protein was conducted in neuronal cells (N2A and hippocampal neurons). The hippocampal cells (5 × 10^4^ cells/cm^2^) were cultured in MEM (Invitrogen) supplemented with 5% horse serum, 5% FBS, 0.5 mM glutamine and penicillin/streptomycin (PS, all from Invitrogen) and was allowed to grow for 24 h prior to testing with a range of concentrations of memantine (1, 5, 10, 20 μM). The memantine-treated cells were then lysed using Cytobuster™ (Novagen) and were subsequently analyzed for endogenous tau protein expression. Bicinchoninic acid assay (BCA) was done to determine the total protein concentration and protein separation was carried out with SDS-PAGE. Western blot analysis followed, using the monoclonal antibodies (1:2500) for tau-1 clone PC1C6 (Millipore, Billerica, MA, USA) and rabbit polyclonal antibody against APP (ab207; ABCAM Company, Cambridge, UK) to detect the target proteins from the cell lysates.

### 4.4. Fermentation of Traditional Chinese Herb

All the *Lactobacillus* spp. used in this study were purchased from Bioresources Collection and Research Center (BCRC), Hsin Tue, Taiwan and were preserved in Lactobacilli MRS broth (DIFCO, Detroit, MI, USA) (−80 °C, with 15% glycerol). To prepare the products of *Lactobacillus* spp. fermented Chinese herb, these *Lactobacillus* spp. were first activated in Lactobacilli MRS broth with 0.05% l-cysteine (100 mL, 37 °C) for 24 h. The Chinese herbs (700 g) were added with the activated *Lactobacillus* spp. and incubated in a 10 liter fermenter with 7 liter culture broth for 24 h at 37 °C. The composition of the culture broth contained: peptone (20 g/L); yeastolate (10 g/L) and glucose (70 g/L) in potassium phosphate buffer pH 7.4.

### 4.5. Preparation of the Fermentation Products

After the Chinese herbs including: *Eleutherococcus senticosus*, *Lycium chinense* Miller, *Panax ginseg*, *Curcuma longa*, *Radix notoginseng* and *Gastrodia elata* were fermented with the *Lactobacillus* spp., the ferments were extracted with 70% alcohol for two days and filtrated twice. The filtrates were collected and concentrated with rotary evaporator and then with lyophilizer to remove remained water. The powders of these *Lactobacillus* spp. fermented Chinese herbs were stored in −20 °C freezer before use.

### 4.6. Animals

All procedures were performed according to the National Institutes of Health Guidelines for the Use of Laboratory Animals and approved by the Institutional Animal Care and Use Committee of Chung Tuan Christian University, Taoyuan, Taiwan. ApoE^−/−^ mice with the C57BL/6 genetic background were provided by National Taiwan University (S.W. Lin). Mice (male, 10 weeks old) were randomly divided into two groups (*n* = 8 per group) and were fed either HFD containing 60% of kilocalories from fat (TestDiet; LabDiet Cat No. 58Y1) or HFD plus *NB34* for 12 weeks. The *NB34* group received 4 mg/kg body weight (bw)/day *NB34* via a gavage of gastric tube for 12 weeks, with dose adjustment weekly according to body weight.

### 4.7. Morris Water Maze (MWM) Task

The spatial learning performance of the control or *NB34* treated ApoE^−/−^ mice was assessed in a white circular water tank (diameter 120 cm, depth 45 cm) filled with tap water (25 ± 0.5 °C). The water tank was located in a test room that contained several cues around the maze and remained unchanged during the test. Each mouse was subjected to a series trial, 4 trials per day. For each trial, the place where the mouse was put in the water differed with four different positions. If the mouse could not find the platform within 60 s, it was guided to the platform with a sieve, and after it was on the platform for 20 s it was then put into its cage. After completing the 5 days learning of the MWM, memory recall was determined by a probe test. This probe test was performed 24 h after the acquisition, and measured the ability to consolidate spatial memories. For the acquisition trial the latency period of these control or NB34 treated mice to reach the platform were recorded and compared. For the probe trial, the platform was removed from the water tank, and the time periods the mice needed to swim in the four quarters were recorded and analysed using a video-tracking software (Ethovision XT 7, Noldus Information Technology, Wageningen, The Netherlands). Water maze search strategy analysis was performed as described previously [51,52]. The search strategies were analyzed for the first of the four trials of the Morris Water Maze for six days. The percentage of each strategy in each group was calculated. Swim strategies were characterized as spatial, systematic, or looping, and representative strategies are provided (Figure 7C).

### 4.8. Data Analysis

All data were presented as the means ± SEM. Statistical comparisons were performed by paired or unpaired Student’s *t*-tests, and one-way analysis of variance (ANOVA) or two way ANOVA for repeated experiments followed by Fisher’s protected least significant different test. *p* < 0.05 was considered to indicated a statistically significant difference.
